# Prospective longitudinal study of subcortical brain volumes in individuals at high familial risk of mood disorders with or without subsequent onset of depression

**DOI:** 10.1016/j.pscychresns.2015.12.009

**Published:** 2016-02-28

**Authors:** Martina Papmeyer, Jessika E. Sussmann, Tiffany Stewart, Stephen Giles, John G. Centola, Vasileios Zannias, Stephen M. Lawrie, Heather C. Whalley, Andrew M. McIntosh

**Affiliations:** a Division of Psychiatry, University of Edinburgh, Royal Edinburgh Hospital, Morningside Park, Edinburgh EH10 5HF, United Kingdom; b Division of Systems Neuroscience of Psychopathology, Translational Research Center, University Hospital of Psychiatry, University of Bern, Bolligenstrasse 111, 3000 Bern 60, Switzerland

**Keywords:** Magnetic resonance imaging, Neuroimaging, Major depressive disorder, Bipolar disorder

## Abstract

Subcortical volumetric brain abnormalities have been observed in mood disorders. However, it is unknown whether these reflect adverse effects predisposing to mood disorders or emerge at illness onset. Magnetic resonance imaging was conducted at baseline and after two years in 111 initially unaffected young adults at increased risk of mood disorders because of a close family history of bipolar disorder and 93 healthy controls (HC). During the follow-up, 20 high-risk subjects developed major depressive disorder (HR-MDD), with the others remaining well (HR-well). Volumes of the lateral ventricles, caudate, putamen, pallidum, thalamus, hippocampus and amygdala were extracted for each hemisphere. Using linear mixed-effects models, differences and longitudinal changes in subcortical volumes were investigated between groups (HC, HR-MDD, HR-well). There were no significant differences for any subcortical volume between groups controlling for multiple testing. Additionally, no significant differences emerged between groups over time. Our results indicate that volumetric subcortical brain abnormalities of these regions using the current method appear not to form familial trait markers for vulnerability to mood disorders in close relatives of bipolar disorder patients over the two-year time period studied. Moreover, they do not appear to reduce in response to illness onset at least for the time period studied.

## Highlights

• Longitudinal study of subcortical brain volumes in high-risk of mood disorder group.
• High-risk subjects developed depression or remained well during two-year follow-up.
• The subcortical volumes are not associated with familial risk of mood disorders.
• The subcortical volumes do not increase in response to depression onset.

## 1 Introduction

The common psychopathological symptom in mood disorders such as bipolar disorder (BD) and major depressive disorder (MDD) is a disturbance in the person's mood (World Health Organization, 2004). Several lines of evidence indicate that there is an overlap in the pathophysiological mechanisms underlying the pathogenesis of BD and MDD. First of all, mood disorders commonly aggregate within families, with first-degree relatives of BD patients having a 10-fold increased risk of BD and a 3-fold excess risk of MDD as compared to the general population (Smoller and Finn, 2003). Moreover, moderate to high heritability estimates have been found and recent findings indicate that both conditions share underlying genetic risk factors (Craddock, 2006, Cross-Disorder Group of the Psychiatric Genomics Consortium, 2013, McGuffin et al., 2003, Schulze et al., 2014).

Using region-of-interest (ROI) manual tracing, semi-automated segmentation methods or voxel-based morphometry analyses, structural magnetic resonance imaging (MRI) studies have repeatedly shown that gray matter abnormalities in subcortical brain regions are associated with mood disorders (Beyer and Krishnan, 2002, Konarski et al., 2008, Savitz and Drevets, 2009). Most consistently, meta- and mega-analyses have detected a significant enlargement of the lateral ventricles in both MDD and BD patients (Arnone et al., 2009, Hallahan et al., 2011, Kempton et al., 2008, Kempton et al., 2011, McDonald et al., 2004). It has been hypothesized that this ventricular abnormality may reflect medial temporal lobe, lateral prefrontal cortex or basal ganglia volume reductions (Savitz and Drevets, 2009). Furthermore, reduced basal ganglia volumes of the caudate, putamen and pallidum have been repeatedly found in MDD (Arnone et al., 2012, Bora et al., 2012, Kempton et al., 2011, Koolschijn et al., 2009), while findings in BD have been largely inconsistent, with volumes being either increased (Arnone et al., 2009, Hallahan et al., 2011) or within the normal range (Kempton et al., 2008, McDonald et al., 2004). In MDD (Arnone et al., 2012, Kempton et al., 2011, Koolschijn et al., 2009) but not BD patients (Arnone et al., 2009, Hallahan et al., 2011, Kempton et al., 2008), the thalamus and hippocampus have been found to be reduced. Of note, some recent individual voxel-based morphometry or segmentation-based studies did also detect reduced hippocampal gray matter volumes in BD patients (Quigley et al., 2015, Redlich et al., 2014). Finally, there is some evidence for volume reductions of the amygdala in both conditions (Bora et al., 2012, Hallahan et al., 2011). However, the majority of meta-analyses have not detected amygdala volume abnormalities in MDD or BD patients (Arnone et al., 2009, Arnone et al., 2012, Kempton et al., 2008, Koolschijn et al., 2009, McDonald et al., 2004). Importantly, a large heterogeneity in findings has been observed for the amygdala, with smaller amygdala volumes being particularly observed in paediatric, adolescent and young adult MDD or BD subjects and larger volumes being most frequently found in older age for both conditions (Konarski et al., 2008, Schmaal et al., 2015). Moreover, research indicates that family history for depression and gender impact on the volume of the amygdala, providing a further rationale for the heterogeneous findings concerning amygdala volumes in MDD (Saleh et al., 2012). In detail, a positive family history for depression in female but not male MDD patients has been associated with smaller amygdala volumes, whereas depression without a positive family history has been associated with increased amygdala volumes (Saleh et al., 2012).

The subcortical brain regions outlined are of particular interest for the pathogenesis of mood disorders as they are closely involved in affect regulation and emotion processing – functions clearly disturbed in mood disorders (Phillips et al., 2003a, Phillips et al., 2003b). The amygdala, ventral striatum, thalamus and hippocampus are selectively interconnected with distinct prefrontal and anterior cingulate brain regions in two relatively discrete neural systems. Accordingly, it has been postulated that dysfunction in specific components of these neural systems could be centrally involved in the mood dysregulation observed in BD and MDD (Phillips et al., 2003b).

The aetiology of the commonly observed subcortical brain abnormalities in mood disorders remains relatively unknown as most brain imaging research has focussed on brain structure in individuals already affected by the disease. Accordingly, they cannot provide information whether volumetric brain abnormalities represent early neurodevelopment disturbances that are present prior to illness onset, emerge in response to pathological processes occurring close to illness onset, or whether they represent the effects of chronic illness or medication. Neuroimaging investigations of individuals at high familial risk of mood disorders may help to detect structural brain abnormalities associated with familial vulnerability, unconfounded by illness and medication effects. However, only few studies have examined subcortical gray matter volumes in unaffected close relatives of BD patients as yet. These have yielded largely heterogeneous results (Nery et al., 2013), with a recent meta-analysis indicating no evidence towards subcortical gray matter abnormalities (Fusar-Poli et al., 2012). It appears plausible that the inconsistency in findings, in particular with regard to the amygdala, is partly related to factors that have been found to influence subcortical brain volumes in affected patients such as age and gender as outlined earlier. To the best of our knowledge, no study has yet examined prospectively and longitudinally the effects of the presence of familial risk on subcortical gray matter volumes.

The Bipolar Family Study allows investigating the time course of volumetric subcortical brain abnormalities in mood disorders and their association with familial risk and illness onset. We have previously shown that reduced right parahippocampal and fusiform gyrus thickness across time as well as progressive cortical thinning in the left inferior frontal and precentral gyri over time are familial vulnerability marker for mood disorders (Papmeyer et al., 2015). Moreover, we found the onset of depression to be associated with abnormal left inferior frontal and precentral thickness development over time in this study cohort (Papmeyer et al., 2015). However, subcortical brain volumes of our study cohort have not been studied as yet.

Based on the literature review presented above, we compared the volumes of the lateral ventricles, caudate, putamen, pallidum, thalamus, hippocampus and amygdala for each hemisphere over a two-year time interval between three groups: individuals at high risk of mood disorders who were not affected by mood disorders at study entry but had an onset of MDD during the follow-up time (HR-MDD), individuals at high risk who remained unaffected by mood disorders during the identical time period (HR-well), and healthy control individuals (HC). All study participants were recruited during late adolescence or early adulthood. Given the relatively young age of our participants and the observed associations between small amygdala volumes and a positive family history for depression and young age in mood disorders patients, we hypothesised that reduced volume of the amygdala is associated with familial risk for mood disorders and is thus present in unaffected close BD relatives. Moreover, we hypothesised that subcortical regions commonly reduced in mood disorders patients reduce in volume progressively in the two-year follow-up period prior to illness and that the onset of MDD results in more pronounced volume reductions as compared to high-risk individuals who remain unaffected by the disorder.

## 2 Methods

### 2.1 Participants

All participants for this research project were recruited as part of the Bipolar Family Study (Sprooten et al., 2011, Whalley et al., 2011). Individuals at high risk of mood disorders because of a close family history of BD were identified via affected relatives. They were considered at increased risk of mood disorders because of the known cross-over of risk between BD and MDD. Across Scotland, psychiatrists referred patients to the study with a primary diagnosis of BD, type I. To confirm a diagnosis in affected subjects, the Operational Criteria Symptom Checklist (McGuffin et al., 1991) using information from clinical case notes and the Structured Clinical Interview for DSM-IV Axis-I Disorders (First et al., 1996) were employed. The BD patients were asked to identify close family members between 16 and 25 years of age. Following informed consent, unaffected individuals with at least one first degree, or two second degree relatives with type I BD were invited to take part in the study. All high risk of mood disorders participants were interviewed to confirm a lifetime absence of mood disorders or schizophrenia and to ensure that they did not meet any exclusion criteria outlined below.

Healthy unrelated control subjects with no personal or family history of BD were identified from the social contacts of the high-risk participants and group-matched for age, sex and premorbid intelligence estimated with the National Adult Reading Test (Nelson, 1982). To screen for Axis-I disorders, the Structured Clinical Interview for DSM-IV Axis-I Disorders (First et al., 1996) was used.

The exclusion criteria for all study groups at baseline included a personal history of MDD, mania or hypomania, psychosis, or any major neurological or psychiatric disorder, substance dependence, learning disability, head injury that included loss of consciousness and any contraindications to MRI.

Approximately two years after study entry, all study participants were invited for a follow-up appointment. All subjects provided written informed consent and the study was approved by the Research Ethics Committee for Scotland.

### 2.2 Clinical assessment

Clinical assessments were carried out on the same day as the first and second MRI scan. The average time interval in years between the two visits was 2.13 (*SD* 0.22), 2.15 (*SD* 0.22), 2.10 (*SD* 0.13) for the HC, HR-well and HR-MDD group. To determine the diagnostic status of consenting subjects who did not return for a second visit, the National Health Service was contacted. Two experienced psychiatrists conducted the clinical interviews (AMM, JES). On the basis of the follow-up clinical assessment or information from case notes, individuals at high risk of mood disorders were grouped into those who remained well (HR-well), and those who developed MDD after study entry (HR-MDD). Current manic and depressive symptoms were determined at baseline and follow-up assessments with the Young Mania Rating Scale (Young et al., 2000) and Hamilton Depression Rating Scale (Hamilton, 1960).

### 2.3 Magnetic resonance imaging acquisition

Imaging at both time points was carried out at the Brain Imaging Research Centre (BIRC) for Scotland on a GE 1.5 T Signa scanner (GE Medical, Milwaukee, USA). The T_1_ sequence was a coronal gradient echo sequence with magnetisation preparation (MPRAGE) and yielded 180 contiguous 1.2 mm coronal slices (TI=500 ms; TE=4 ms; matrix=192×192; flip angle=8°).

### 2.4 Subcortical volume segmentations

The T1 images were processed using the volume-based stream in FreeSurfer version 5.1.0 (http://surfer.nmr.mgh.harvard.edu), described in detail by Fischl et al. (2002). In short, intensity normalisation was conducted, followed by removal of non-brain tissue using a hybrid watershed/surface deformation procedure. Next, neuroanatomical labels were assigned to each voxel in an MRI volume based on probabilistic information derived from a manually labelled training set. The accuracy of the final subcortical brain segmentations was visually inspected and edited where needed according to the standard FreeSurfer guidelines (http://surfer.nmr.mgh.harvard.edu/fswiki/Edits) by a single rater, blind to diagnostic status (MP).

**Fig. 1:**
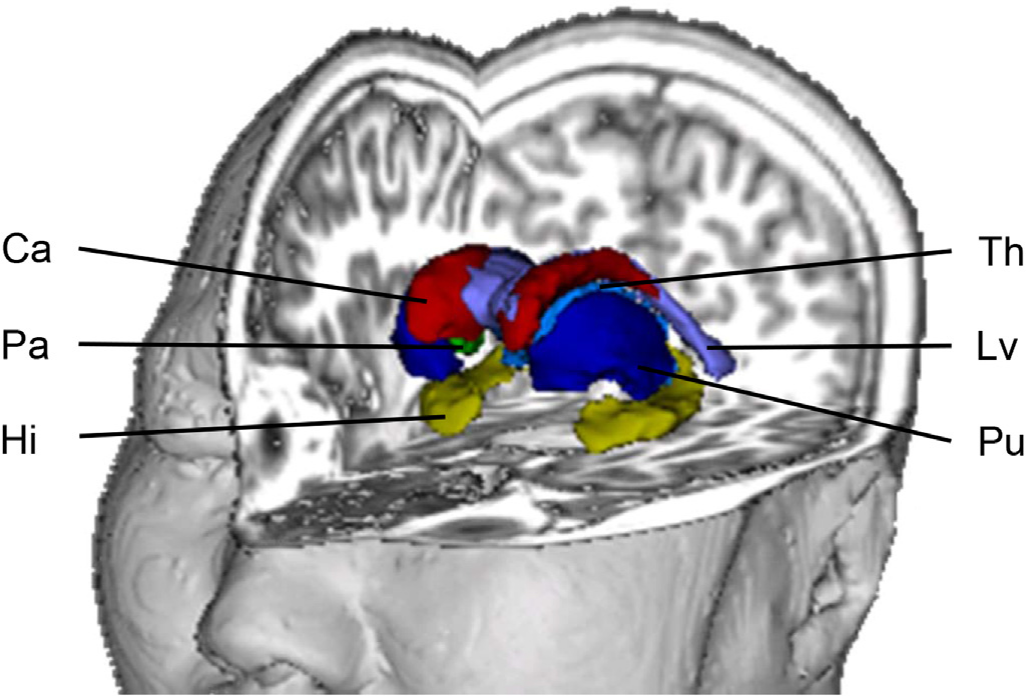
Subcortical regions of interest. Three-dimensional representation of the subcortical regions of interest extracted with FreeSurfer. Not shown: amygdala. Abbreviations: Ca, caudate; Hi, hippocampus; Lv, lateral ventricles; Pa, pallidum; Pu, putamen; Th, thalamus.

The following regions of interest (ROI) were extracted for each hemisphere (Fig. 1): lateral ventricles, caudate, putamen, pallidum, hippocampus, thalamus, amygdala. We decided to compare the volumes of these subcortical brain regions separately for each hemisphere since some findings in unaffected relatives of BD patients or first-episode MDD patients point towards specific lateralisation effects (Frodl et al., 2002, Nery et al., 2013). In addition, intra-cranial volume of the whole brain was extracted to serve as a covariate.

### 2.5 Statistical analyses

The statistical analyses were conducted using SPSS version 19 (http://www.spss.com), except for False Discovery Rate (FDR) corrections (Benjamini and Hochberg, 1995) which were computed using the ‘p.adjust(BH)’ function of the ‘stats’ package in R version 2.13.0 (http://www.r-project.org). Demographic and clinical data analyses were conducted using one-way analyses of variance (ANOVA), chi-squares tests or Kruskal-Wallis tests where appropriate.

Linear mixed-effects models were applied to analyse structural brain differences between groups for each ROI over time. These models have several advantages over repeated measures ANOVAs as they take the correlation structures of repeated measurements nested within participants into account and do not require casewise deletion of missing data so that all available data can be analysed. In the linear mixed-effects model used, the intercept term is entered as a random effect that varies by individual so that intraindividual correlation among the volumetric brain measures of a given subject is taken into account. The following independent variables were used as predictors of subcortical brain volume for the different ROIs: group, time (baseline versus follow-up assessment), group-by-time interaction. Age, sex and intracranial volume served as covariates. Accordingly, differences in subcortical brain volume between the groups across both time points are represented by significant group effects. Differences in subcortical brain volume between baseline and follow-up examination are represented as time effects. Differences in subcortical brain volume development over time between groups are represented as group-by-time interactions.

A statistical significance level of *p*_*FDR*_≤0.05 was selected, fully corrected for multiple comparisons using the Benjamini & Hochberg FDR procedure (Benjamini and Hochberg, 1995). For ease of comparison with future results, we report the original uncorrected p-values (*p*_*uncorrected*_) and whether they survived the FDR threshold. Wherever significant effects were found, pairwise comparisons were conducted between the three groups, with p-values being corrected according to Tukey's “Honest significance difference” method (*p*_*HSD*_≤0.05).

The putative relationship between depressive symptom severity and subcortical brain volumes was examined by calculating the Spearman correlation coefficient between the Hamilton Depression Rating Scale total score and the ROIs for each group. For all analyses, p-values were corrected using the FDR procedure and considered significant when *p*_*FDR*_≤0.05.

To assess potentially confounding effects of medication and familial relatedness of subjects on the volumes of the ROIs, we conducted the following supplemental analyses for significant results: First, we repeated our analyses excluding medicated HR-MDD subjects (*n*=4). Second, we randomly excluded related subjects from the analyses (*n*=2 HC; *n*=17 HR-well; *n*=2 HR-MDD).

## 3 Results

### 3.1 Demographic and clinical characteristics

FreeSurfer processed MRI scans of good quality along with clinical data were available for 114 high-risk individuals at baseline. Two of these individuals had developed BD during the two-year follow-up time and were excluded from all analyses because of their small sample size. In total, 20 high-risk subjects had an onset of MDD during the two-year follow-up period. However, one of them was excluded from baseline analysis due to the unsatisfactory subcortical segmentations of the MRI scan. Accordingly, our analyses included 92 HR-well and 19 HR-MDD subjects at baseline. There were 96 HC subjects that provided MRI data of good quality along with clinical information at baseline. Three of them had an onset of MDD during the follow-up time and were thus excluded from all analyses, resulting in a sample size of 93 HC participants. At follow-up examination, there were 63 HR-well, 20 HR-MDD, and 62 HC subjects with suitable processed MRI scans together with clinical data. Among the HR-MDD participants, four were receiving antidepressant medication at follow-up. Three individuals were taking selective serotonin reuptake inhibitors (1citalopram, 1 fluoxetine, 1 sertraline) and one participant was on a tricyclic antidepressant (lofepramine). The remaining 16 HR-MDD subjects were unmedicated.

No significant differences between the groups emerged in terms of age, gender, handedness, verbal intelligence and Young Mania Rating Scale total score at any assessment point (Table 1). However, there were significant group differences at baseline (*p*≤0.007) and follow-up (*p*≤0.023) for clinical measures of depression using the Hamilton Depression Rating Scale. At baseline, HR-well and HR-MDD participants had significantly higher depression sum scores (*p*≤0.047 and *p*≤0.003, respectively) than HC subjects, with no significant differences between the two high-risk groups. At follow-up, HR-MDD subjects had higher depression sum scores than HC and HR-well individuals (*p*≤0.013 and *p*≤0.010, respectively) as expected, with no significant differences between HC and HR-well individuals.

### 3.2 Subcortical brain volumes

The linear mixed-effects model analyses revealed no significant effects of group after correction for multiple testing. Also, no significant effects of time or group-by-time interactions for any ROI volume were observed after correction for multiple testing (Table 2). Of note, a trend towards a significant group effect was observed for the right caudate (*p*_*uncorrected*_≤0.024), with the HR-MDD group having smaller volumes than the HC (*p*_*HSD*_≤0.024) and the HR-well (*p*_*HSD*_≤0.016) group across time. Also, there was a nominally significant group-by-time interaction for the volume of the left amygdala (*p*_*uncorrected*_≤0.036). Post-hoc analyses revealed that the volume of this structure decreased over time in the HR-well as compared to the HC group that in turn displayed volumetric increases (*p*_*HSD*_≤0.024). No significant interaction effects for the HR-MDD as compared to the HC group (*p*_*HSD*_≤0.504) and the HR-well as compared to HR-MDD participants (*p*_*HSD*_≤0.158) were found. These effects were only significant at nominal level and did not survive FDR correction.

### 3.3 Correlation analysis

The correlation analysis yielded no significant associations between any volume of the ROIs and depression severity as measured with the Hamilton Depression Rating Scale total score (Table 3).

### 3.4 Analysis of potential confounders

To eliminate the potential confounding effects of familial relatedness of some subjects, the longitudinal analyses were repeated, excluding randomly individuals from the same family (Supplemental Table S1). This analysis similarly yielded no significant effect of group, time or group-by-time interaction. Also, when excluding medicated HR-MDD subjects from the analyses, no significant effects were observed (Supplemental Table S2).

## 4 Discussion

To the best of our knowledge, this is one of the first prospective longitudinal studies investigating subcortical brain volumes in familial high-risk of mood disorders individuals who were unaffected at study entry and either had an onset of MDD or remained well during the two-year follow-up time interval. In contrast to our initial hypothesis, we observed no significant volumetric differences between the groups for any ROI using the current approach over a two-year time interval between baseline and follow-up visit. Moreover, no significant effects of time or group-by-time interactions were found. Similarly, the correlation analyses yielded no significant association between any subcortical brain volume and depressive symptom severity. Excluding participants of the same pedigree or medicated individuals did not alter these results.

Of note, a nominal significant group effect for the volume of the right caudate was found, indicating smaller volumes in the HR-MDD as compared to the other two study groups across both time points. Furthermore, a nominal significant group-by-time interaction was observed for the volume of the left amygdala. The interaction was driven by the HR-well group displaying decreasing left amygdala volume over time as compared to the HC group which in turn showed a volumetric increase. Since both findings did not survive correction for multiple testing however, these results should be interpreted with caution and we suggest to study the volumes of the right caudate and left amygdala prospectively in individuals at high familial risk using larger sample sizes to be able to detect even slightest volume abnormalities.

Our finding of unaltered subcortical brain volumes in HR-well subjects as compared to the HC group is in line with a recent meta-analysis that found no evidence towards subcortical brain volume abnormalities in unaffected close relatives of BD patients (of *n*=122–485 HC and *n*=65–246 HR individuals, depending on the brain structure analysed) (Fusar-Poli et al., 2012). Interestingly, the few studies employing structural brain imaging techniques in unaffected individuals with a close family history of MDD have repeatedly detected abnormal hippocampus (Amico et al., 2011, Baare et al., 2010, Rao et al., 2010, Romanczuk-Seiferth et al., 2014) and amygdala (Lupien et al., 2011, Romanczuk-Seiferth et al., 2014) volumes as compared to individuals with no family history of MDD. Accordingly, this may suggest that specific subcortical brain regions are differentially influenced by genetic and environmental risk factors specific to MDD and BD, respectively. This hypothesis would also be in line with a study by Saleh et al. (2012) who observed opposing effects of a positive or negative family history of MDD on the volume of the amygdala in depressed individuals. Taken together, our findings imply that volumetric subcortical brain abnormalities do not constitute a familial trait marker for vulnerability to mood disorders in close BD relatives. However, it may well be the case that subcortical brain abnormalities do serve as a vulnerability marker for mood disorders in individuals exposed to distinct genetic and environmental risk factors. In line with this hypothesis, Opel et al. (2015) detected gray matter reductions in the hippocampus of individuals at high risk of MDD because of the environmental risk factor of childhood maltreatment but not in individuals at high risk of MDD because of a close family history of the disease. By contrast, the familial risk individuals of their study showed gray matter reductions in cortical brain regions, including the insula and orbitofrontal cortex.

The absence of volumetric subcortical brain abnormalities across time in the HR-MDD group and the absence of group-by-time interactions is opposed to cross-sectional meta-analytic and mega-analytic findings that repeatedly detected volumetric abnormalities of the lateral ventricles, basal ganglia, hippocampus and thalamus in patients with mood disorders (Arnone et al., 2009, Arnone et al., 2012, Bora et al., 2012, Hallahan et al., 2011, Kempton et al., 2008, Kempton et al., 2011, Koolschijn et al., 2009, McDonald et al., 2004). Moreover, these findings are partly in contrast to a recent prospective longitudinal study by Whittle et al. (2014) who found volumetric changes in the hippocampus, amygdala and putamen to be associated with an onset of depression in individuals initially presenting with prodromal depressive symptoms. However, it appears likely that this discrepancy in findings is due to systematic differences in the study design. In particular, Whittle et al. (2014) studied young adolescents who were on average only 12 years of age at study entrance and used a longer inter-scan interval of four years. Moreover, individuals were initially presenting with putative prodromal symptoms while our high-risk participants were selected based on a positive family history of BD, with research on other psychiatric conditions like psychosis indicating that structural brain abnormalities are more pronounced in clinical high risk versus familial high risk individuals (Smieskova et al., 2013). Most of all, it appears likely that our high-risk study participants who developed MDD were exposed to distinct genetic and environmental risk factors associated with a family history of BD as compared to individuals with no family history of BD. All in all, our results provide a first hint to suggest that volumetric subcortical brain abnormalities may not predate an onset of MDD or emerge as a consequence of illness-specific mechanisms linked to the onset of the disorder in close relatives of BD patients. Rather, we hypothesize that volumetric abnormalities may only emerge during the course of the disorder. Subcortical brain volumes may also be influenced by the severity or length of illness, the age at illness onset or the length and type of psychopharmacological treatment.

Using functional MRI approaches, our research group has previously shown that the high-risk group at baseline, when all individuals were still unaffected by disease, show abnormal amygdala activation during an executive processing task (Whalley et al., 2011) and that amygdala activation is related to cumulative genetic risk for BD using polygenic risk profiling (Whalley et al., 2012). Moreover, we have shown that HR-MDD as compared to HR-well subjects exhibit increased brain activation in cortico-thalamic-limbic regions encompassing the thalamus prior to illness onset (Whalley et al., 2015, Whalley et al., 2013). The findings of the present study suggest that the observed aberrant brain activity in the high-risk group as whole or the HR-MDD group appears to be unrelated to volumetric structural brain abnormalities in the same brain region.

The strengths of this study are its longitudinal study design, the assessment of brain morphology prior to illness onset and the relatively large sample size of high-risk and control individuals. Another advantage relates to the relatively young age of the participants at study entrance since early adulthood is considered a critical phase for both illness onset as well as neurodevelopmental processes (Giedd, 2004, Kessler et al., 2005). Furthermore, all participants underwent careful clinical assessment at both appointments and medication effects as well as effects of familial relatedness were ruled out. All brain scans were acquired at the same MRI scanner using an identical imaging protocol at both visits and the MRI data processing was conducted in an identical way with thoroughly validated methods.

Nevertheless, some limitations need to be addressed. First, it cannot be ruled out that currently unaffected HR-well subjects may have an onset of MDD in the future. Second, it appears likely that some of the HR-MDD participants may develop BD in the future since longitudinal studies have documented that the majority of high-risk individuals who went on to develop BD themselves had depressive episodes years before conversion (Duffy, 2010, Hillegers et al., 2005). Future follow-up examinations of our study cohort will clarify these aspects. Third, our sample size of 20 HR-MDD subjects is relatively small which diminishes statistical power to detect small-sized volumetric differences between the groups or over time. Fourth, our study groups differed with regard to their depression symptom severity at baseline. However, the median of the Hamilton Depression Rating Scale total score was only 1 in the HR-MDD group, corresponding to only sub-syndromal depression symptoms. Furthermore, no relationship between depression symptom severity and the subcortical brain volumes was observed. Accordingly, it appears unlikely that group-differences in mood at baseline have influenced our results. Fifth, the precise onset of MDD could not be determined so that it remains unknown whether the duration of the depressive episode until the second MRI scan was obtained might have influenced the results. Sixth, the two-year follow-up period may have been relatively short for brain changes to occur at such a magnitude that they are detectable with our sample size. Future follow-up examinations of our study cohort will further explore this aspect. Finally, it has been shown that the reliability of subcortical brain structure segmentations using FreeSurfer is reduced in brain regions that are small in volume such as the amygdala as compared to larger brain structures (Morey et al., 2010).

In summary, no significant volumetric abnormalities of the lateral ventricles, basal ganglia, thalamus, hippocampus and amygdala were detected in individuals at high familial risk of mood disorders, regardless of an onset of MDD or not. Accordingly, it can be concluded that subcortical volumetric brain abnormalities that are often observed in mood disorders only emerge during the course of illness and are not related to the illness onset or enhanced familial risk for the disorder, at least in close BD relatives. These findings advance our understanding of the neuropathological processes underlying mood disorders. Future prospective longitudinal studies are required that particularly investigate the course of subcortical brain volume development before and after the onset of depression using longer time intervals.

## Contributors

Author AMM designed the study and wrote the protocol. Authors AMM and JES conducted psychiatric interviews with the participants. Author MP processed the MRI scans with FreeSurfer, conducted statistical analyses and wrote the manuscript. All authors contributed to and have approved the final manuscript.

## Conflicts of interest

MP was supported by a studentship from the Medical Research Council. JES is supported by a Clinical Research Training Fellowship from the Wellcome Trust. HCW is supported by a College Fellowship from the University of Edinburgh and a JMAS SIM fellowship from the Royal College of Physicians of Edinburgh. AMM was supported by the Health Foundation through a Clinician Scientist Fellowship (Ref: 2268/4295), by the Brain and Behaviour Research Foundation through a NARSAD Independent Investigator Award and by a Scottish Funding Council Senior Clinical Fellowship. SML, HCW and AMM have received financial support from Pfizer (formerly Wyeth) in relation to imaging studies of people with schizophrenia and bipolar disorder. SML and AMM have done consultancy work for Roche Pharmaceuticals. SML has also received honoraria for lectures, chairing meetings, and consultancy work from Janssen in connection with brain imaging and therapeutic initiatives for psychosis. The authors MP, JES, TS, SG, JGC and VZ have no competing interests to declare.

## Figures and Tables

**Table 1:** Demographic and clinical characteristics.

	**Baseline**					**Follow-up**				
	**HC** (n=93)	**HR-well** (n=92)	**HR-MDD** (n=19)	**Statistics**		**HC** (n=62)	**HR-well** (n=63)	**HR-MDD** (n=20)	**Statistics**	
	Mean (SD)	Mean (SD)	Mean (SD)	F/χ2	p	Mean (SD)	Mean (SD)	Mean (SD)	F/χ2	p
**Age** (years)	21.01 (2.45)	21.20 (2.88)	21.10 (2.82)	0.13	0.88	22.82 (2.73)	23.71 (2.84)	23.33 (2.98)	1.77	0.17
**Gender** (M:F)	40:53	44:48	9:10	0.46	0.80	21:41	29:34	7:13	2.12	0.35
**Handedness** (Right:other)	88:5	81:11	19:0	5.54	0.24	61:1	57:6	20:0	5.43	0.07
**NART IQ**	110.31 (8.00)	108.39 (9.37)	107.26 (6.80)	1.64	0.20	–	–	–	–	–
**Time** (years)	2.13 (0.22)	2.15 (0.22)	2.10 (0.13)	0.20	0.82	–	–	–	–	–
**HAM-D**^b^	0 (1)	0 (2)	1 (5)	9.79	0.01^a^	1 (3)	1 (2)	5 (12)	7.59	0.02^a^
**YMRS**^b^	0 (0)	0 (0)	0 (0)	3.48	0.18	0 (0)	0 (1)	0 (0)	0.79	0.68

Abbreviations: F, female; HAM-D, Hamilton Depression Rating Scale; HC, unaffected healthy control subjects; HR, high risk; HR-MDD, individuals at high risk for mood disorders who were well at baseline but developed major depressive disorder during the follow-up period; HR-well, individuals at high risk of mood disorders who were well at baseline and remained well during the follow-up period; M, male; NART, National Adult Reading Test; Time, Time between baseline and follow-up assessment; YMRS, Young Mania Rating Scale.

a Significant effect.

b Kruskal-Wallis test, median and interquartile presented for skewed variables.

**Table 2:** Longitudinal analysis of regional subcortical volumes.

	**HC**		**HR-well**		**HR-MDD**		**Statistics**					
	**Baseline** (n=93)	**Follow-up** (n=62)	**Baseline** (n=92)	**Follow-up** (n=63)	**Baseline** (n=19)	**Follow-up** (n=20)	**Group effect**		**Time effect**		**GroupXTime**	
**Region**	Mean (SD)	Mean (SD)	Mean (SD)	Mean (SD)	Mean (SD)	Mean (SD)	F	p	F	p	F	p
L lat ventricle	6.69 (2.05)	6.98 (2.74)	7.17 (2.50)	7.28 (2.69)	5.71 (2.45)	5.57 (2.65)	1.25	0.29	1.80	0.18	0.62	0.54
R lat ventricle	6.04 (2.44)	6.27 (2.06)	6.48 (2.20)	6.56 (2.38)	5.40 (2.83)	5.46 (2.01)	0.79	0.46	0.88	0.35	0.13	0.88
L caudate	3.68 (0.43)	3.70 (0.53)	3.71 (0.44)	3.60 (0.45)	3.53 (0.46)	3.44 (0.51)	1.74	0.18	2.03	0.16	1.27	0.28
R caudate	3.83 (0.45)	3.89 (0.59)	3.88 (0.47)	3.83 (0.49)	3.62 (0.47)	3.51 (0.50)	3.81	0.02	0.48	0.49	0.92	0.40
L putamen	5.91 (0.78)	6.02 (0.83)	6.03 (0.72)	6.01 (0.82)	6.09 (0.77)	5.77 (0.83)	0.11	0.90	1.22	0.27	2.42	0.09
R putamen	5.71 (0.67)	5.81 (0.71)	5.72 (0.61)	5.69 (0.72)	5.82 (0.65)	5.40 (0.79)	0.45	0.64	2.34	0.13	2.52	0.08
L pallidum	1.94 (0.29)	1.93 (0.29)	1.87 (0.29)	1.88 (0.33)	1.94 (0.29)	1.90 (0.27)	1.27	0.28	0.19	0.66	0.26	0.78
R pallidum	1.86 (0.28)	1.85 (0.32)	1.83 (0.29)	1.79 (0.33)	1.94 (0.29)	1.83 (0.25)	1.04	0.36	2.93	0.09	0.58	0.56
L thalamus	6.58 (0.75)	6.60 (0.66)	6.56 (0.73)	6.38 (0.76)	6.66 (0.75)	6.39 (0.74)	0.88	0.42	2.98	0.09	1.42	0.24
R thalamus	6.62 (0.67)	6.68 (0.70)	6.56 (0.73)	6.48 (0.79)	6.75 (0.76)	6.55 (0.79)	1.10	0.33	0.85	0.36	1.04	0.36
L hippocampus	3.50 (0.45)	3.47 (0.49)	3.49 (0.46)	3.41 (0.40)	3.61 (0.54)	3.44 (0.53)	0.37	0.69	2.64	0.11	0.28	0.76
R hippocampus	3.58 (0.46)	3.65 (0.52)	3.56 (0.47)	3.47 (0.38)	3.65 (0.59)	3.38 (0.54)	1.58	0.21	3.33	0.07	2.54	0.08
L amygdala	1.77 (0.32)	1.83 (0.33)	1.79 (0.35)	1.67 (0.27)	1.79 (0.34)	1.72 (0.23)	1.55	0.22	1.14	0.29	3.40	0.04
R amygdala	1.96 (0.30)	1.97 (0.30)	1.92 (0.33)	1.92 (0.27)	2.06 (0.33)	1.91 (0.26)	1.31	0.27	1.20	0.28	1.12	0.33

Volumes are measured in cm^3^, p-values are presented uncorrected for multiple comparison. Abbreviations: HC, unaffected healthy control subjects; HR-MDD, individuals at high risk for mood disorders who were well at baseline but developed major depressive disorder during the follow-up period; HR-well, individuals at high risk of mood disorders who were well at baseline and remained well during the follow-up period; L, left; lat, ateral; R, right, SD, standard deviation.

**Table 3:** Correlation between regional subcortical volumes and severity of depressive symptoms.

	**HC** (n=93)		**HR-well** (n=92)		**HR-MDD** (n=19)	
**Region**	R	p	R	p	R	p
L lat ventricle	−0.06	0.71	0.10	0.51	0.22	0.44
R lat ventricle	−0.19	0.43	0.13	0.37	0.20	0.48
L caudate	−0.11	0.47	0.27	0.06	0.04	0.88
R caudate	−0.13	0.40	0.27	0.06	0.02	0.95
L putamen	−0.16	0.29	0.05	0.74	0.02	0.94
R putamen	−0.15	0.31	0.30	0.04	−0.10	0.72
L pallidum	−0.05	0.75	0.28	0.05	0.19	0.51
R pallidum	−0.20	0.18	0.13	0.36	0.31	0.26
L thalamus	−0.11	0.47	0.37	0.01	0.11	0.69
R thalamus	−0.18	0.23	0.07	0.64	0.22	0.43
L hippocampus	−0.06	0.71	0.19	0.19	0.15	0.59
R hippocampus	−0.17	0.26	0.31	0.03	0.28	0.32
L amygdala	0.02	0.91	−0.05	0.74	−0.07	0.80
R amygdala	−0.12	0.48	0.03	0.86	0.37	0.17

p-values are presented uncorrected for multiple comparison. Abbreviations: HC, unaffected healthy control subjects; HR-MDD, individuals at high risk for mood disorders who were well at baseline but developed major depressive disorder during the follow-up period; HR-well, individuals at high risk of mood disorders who were well at baseline and remained well during the follow-up period; L, left; lat, lateral; R, right.

## Footnotes

Appendix A Supplementary data associated with this article can be found in the online version at http://dx.doi:10.1016/j.pscychresns.2015.12.009.
