# Supplementary material for: Prospective longitudinal study of subcortical brain volumes in individuals at high familial risk of mood disorders with or without subsequent onset of depression
Source: Psychiatry Res. 2016 Feb 28;248:119–25. doi: 10.1016/j.pscychresns.2015.12.009 (PMC4834463; doi:10.1016/j.pscychresns.2015.12.009)
Supplement: Supplementary file 1 — Supplementary material [file mmc1.docx]

**S1 Table. Longitudinal analysis of subcortical brain volumes in unrelated subjects.**

|  | **HC** | | **HR-well** | | **HR-MDD** | | **Statistics** | | | | | |
| --- | --- | --- | --- | --- | --- | --- | --- | --- | --- | --- | --- | --- |
|  | **Baseline**  (n=93) | **Follow-up**  (n=62) | **Baseline**  (n=92) | **Follow-up**  (n=63) | **Baseline**  (n=19) | **Follow-up**  (n=20) | **Group effect** | | **Time effect** | | **GroupXTime** | |
| **Region** | Mean (SD) | Mean (SD) | Mean (SD) | Mean (SD) | Mean (SD) | Mean (SD) | F | p | F | p | F | p |
| L lat ventricle | 6.68 (2.09) | 6.99 (2.07) | 7.07 (2.78) | 7.08 (2.38) | 5.69 (2.79) | 5.59 (2.33) | 0.91 | 0.40 | 1.20 | 0.28 | 0.73 | 0.48 |
| R lat ventricle | 6.08 (2.46) | 6.27 (2.31) | 6.52 (2.43) | 6.47 (2.35) | 5.40 (1.89) | 5.46 (2.19) | 0.62 | 0.54 | 0.52 | 0.47 | 0.28 | 0.75 |
| L caudate | 3.68 (0.43) | 3.70 (0.53) | 3.69 (0.47) | 3.57 (0.43) | 3.54 (0.47) | 3.43 (0.52) | 1.53 | 0.22 | 2.75 | 0.10 | 1.65 | 0.20 |
| R caudate | 3.83 (0.45) | 3.89 (0.59) | 3.86 (0.49) | 3.78 (0.48) | 3.62 (0.48) | 3.51 (0.51) | 2.89 | 0.06 | 0.92 | 0.34 | 1.25 | 0.29 |
| L putamen | 5.92 (0.79) | 6.02 (0.82) | 6.04 (0.74) | 5.91 (0.74) | 6.07 (0.79) | 5.75 (0.84) | 0.01 | 0.99 | 2.50 | 0.12 | 3.22 | 0.04 |
| R putamen | 5.71 (0.67) | 5.81 (0.70) | 5.73 (0.64) | 5.62 (0.70) | 5.80 (0.66) | 5.36 (0.79) | 0.84 | 0.45 | 3.67 | 0.06 | 2.89 | 0.06 |
| L pallidum | 1.94 (0.29) | 1.93 (0.29) | 1.87 (0.29) | 1.84 (0.34) | 1.95 (0.29) | 1.89 (0.27) | 2.08 | 0.13 | 0.98 | 0.33 | 0.32 | 0.72 |
| R pallidum | 1.86 (0.28) | 1.85 (0.32) | 1.82 (0.30) | 1.73 (0.30) | 1.95 (0.29) | 1.83 (0.26) | 2.64 | 0.07 | 5.01 | 0.03 | 1.05 | 0.35 |
| L thalamus | 6.58 (0.76) | 6.60 (0.66) | 6.58 (0.73) | 6.34 (0.70) | 6.69 (0.76) | 6.32 (0.69) | 1.08 | 0.34 | 4.81 | 0.03 | 2.16 | 0.12 |
| R thalamus | 6.62 (0.68) | 6.68 (0.70) | 6.56 (0.71) | 6.38 (0.72) | 6.77 (0.78) | 6.51 (0.78) | 2.13 | 0.12 | 2.21 | 0.14 | 1.92 | 0.15 |
| L hippocampus | 3.50 (0.45) | 3.46 (0.49) | 3.48 (0.46) | 3.38 (0.39) | 3.59 (0.55) | 3.41 (0.53) | 0.50 | 0.61 | 2.95 | 0.09 | 0.34 | 0.71 |
| R hippocampus | 3.59 (0.46) | 3.65 (0.52) | 3.56 (0.48) | 3.44 (0.37) | 3.65 (0.61) | 3.33 (0.52) | 2.19 | 0.12 | 4.52 | 0.04 | 2.88 | 0.06 |
| L amygdala | 1.76 (0.32) | 1.83 (0.33) | 1.79 (0.34) | 1.67 (0.27) | 1.82 (0.31) | 1.73 (0.23) | 1.34 | 0.26 | 1.46 | 0.23 | 3.40 | 0.04 |
| R amygdala | 1.96 (0.30) | 1.97 (0.30) | 1.91 (0.33) | 1.90 (0.27) | 2.04 (0.33) | 1.90 (0.27) | 1.55 | 0.22 | 1.15 | 0.26 | 0.82 | 0.44 |

Volumes are measured in cm^3^, p-values are presented uncorrected for multiple comparison. Abbreviations: HC, unaffected healthy control subjects; HR-MDD, individuals at high risk for mood disorders who were well at baseline but developed major depressive disorder during the follow-up period; HR-well, individuals at high risk of mood disorders who were well at baseline and remained well during the follow-up period; L, left; lat, lateral; R, right; SD, standard deviation.
