# Supplementary material for: Prospective longitudinal study of subcortical brain volumes in individuals at high familial risk of mood disorders with or without subsequent onset of depression
Source: Psychiatry Res. 2016 Feb 28;248:119–25. doi: 10.1016/j.pscychresns.2015.12.009 (PMC4834463; doi:10.1016/j.pscychresns.2015.12.009)
Supplement: Supplementary file 2 — Supplementary material [file mmc2.docx]

**S2 Table. Longitudinal analysis of subcortical brain volumes in unmedicated subjects.**

|  | **HC** | | **HR-well** | | **HR-MDD** | | **Statistics** | | | | | |
| --- | --- | --- | --- | --- | --- | --- | --- | --- | --- | --- | --- | --- |
|  | **Baseline**  (n=93) | **Follow-up**  (n=62) | **Baseline**  (n=92) | **Follow-up**  (n=63) | **Baseline**  (n=19) | **Follow-up**  (n=20) | **Group effect** | | **Time effect** | | **GroupXTime** | |
| **Region** | Mean (SD) | Mean (SD) | Mean (SD) | Mean (SD) | Mean (SD) | Mean (SD) | F | p | F | p | F | p |
| L lat ventricle | 6.68 (2.09) | 6.99 (2.07) | 7.07 (2.78) | 7.08 (2.38) | 5.87 (2.60) | 5.83 (2.23) | 0.86 | 0.43 | 2.01 | 0.16 | 0.37 | 0.69 |
| R lat ventricle | 6.08 (2.46) | 6.27 (2.31) | 6.52 (2.43) | 6.47 (2.35) | 5.44 (1.75) | 5.63 (2.28) | 0.66 | 0.52 | 0.91 | 0.34 | 0.09 | 0.91 |
| L caudate | 3.68 (0.43) | 3.70 (0.53) | 3.69 (0.47) | 3.57 (0.43) | 3.59 (0.44) | 3.45 (0.45) | 1.36 | 0.26 | 2.21 | 0.14 | 1.35 | 0.26 |
| R caudate | 3.83 (0.45) | 3.89 (0.59) | 3.86 (0.49) | 3.78 (0.48) | 3.70 (0.44) | 3.50 (0.42) | 2.78 | 0.06 | 1.01 | 0.32 | 1.28 | 0.28 |
| L putamen | 5.92 (0.79) | 6.02 (0.82) | 6.04 (0.74) | 5.91 (0.74) | 6.21 (0.75) | 5.73 (0.67) | 0.12 | 0.89 | 1.92 | 0.17 | 2.92 | 0.06 |
| R putamen | 5.71 (0.67) | 5.81 (0.70) | 5.73 (0.64) | 5.62 (0.70) | 5.96 (0.60) | 5.57 (0.58) | 0.25 | 0.78 | 1.81 | 0.18 | 2.08 | 0.13 |
| L pallidum | 1.94 (0.29) | 1.93 (0.29) | 1.87 (0.29) | 1.84 (0.34) | 1.96 (0.29) | 1.88 (0.28) | 1.25 | 0.29 | 0.55 | 0.46 | 0.54 | 0.58 |
| R pallidum | 1.86 (0.28) | 1.85 (0.32) | 1.82 (0.30) | 1.73 (0.30) | 1.97 (0.30) | 1.81 (0.23) | 1.10 | 0.34 | 4.40 | 0.04 | 1.16 | 0.32 |
| L thalamus | 6.58 (0.76) | 6.60 (0.66) | 6.58 (0.73) | 6.34 (0.70) | 6.75 (0.67) | 6.38 (0.67) | 0.94 | 0.39 | 3.97 | 0.05 | 1.79 | 0.17 |
| R thalamus | 6.62 (0.68) | 6.68 (0.70) | 6.56 (0.71) | 6.38 (0.72) | 6.87 (0.67) | 6.50 (0.63) | 1.22 | 0.30 | 1.98 | 0.16 | 1.72 | 0.18 |
| L hippocampus | 3.50 (0.45) | 3.46 (0.49) | 3.48 (0.46) | 3.38 (0.39) | 3.60 (0.55) | 3.45 (0.56) | 0.34 | 0.72 | 1.81 | 0.18 | 0.14 | 0.87 |
| R hippocampus | 3.59 (0.46) | 3.65 (0.52) | 3.56 (0.48) | 3.44 (0.37) | 3.67 (0.60) | 3.34 (0.56) | 1.65 | 0.20 | 4.49 | 0.04 | 3.14 | 0.05 |
| L amygdala | 1.76 (0.32) | 1.83 (0.33) | 1.79 (0.34) | 1.67 (0.27) | 1.76 (0.37) | 1.72 (0.19) | 1.61 | 0.20 | 0.72 | 0.40 | 3.38 | 0.04 |
| R amygdala | 1.96 (0.30) | 1.97 (0.30) | 1.91 (0.33) | 1.90 (0.27) | 2.02 (0.34) | 1.90 (0.25) | 1.07 | 0.35 | 0.68 | 0.41 | 0.67 | 0.51 |

Volumes are measured in cm^3^, p-values are presented uncorrected for multiple comparison. Abbreviations: HC, unaffected healthy control subjects; HR-MDD, individuals at high risk for mood disorders who were well at baseline but developed major depressive disorder during the follow-up period; HR-well, individuals at high risk of mood disorders who were well at baseline and remained well during the follow-up period; L, left; lat, lateral; R, right ; SD, standard deviation.
